# Development and Performance Evaluation of a Smart Upper-Limb Rehabilitation Exercise Device

**DOI:** 10.3390/s24020659

**Published:** 2024-01-19

**Authors:** Bogja Jeoung, Muncheong Choi, Alchan Kim

**Affiliations:** 1 Department of Exercise Rehabilitation, Gachon University, Incheon-shi 21936, Republic of Korea; bogja05@gachon.ac.kr; 2 Exercowork, Co., Ltd., Hanam 12939, Republic of Korea; muncheong.choi@gmail.com; 3 Department of Sports and Technology, Seokyeong University, Seoul 02173, Republic of Korea

**Keywords:** disabilities, isokinetic contractions, isometric strength measurement, musculoskeletal rehabilitation, rehabilitation exercise device, upper-limb rehabilitation

## Abstract

User-friendly rehabilitation medical devices can enhance health and the quality of life through the convergence of information communication and medical technology. Muscle contraction enables bodily movement, and the assessment of muscle strength is crucial. Muscle contraction includes isometric, isotonic, and isokinetic types. Many individuals with physical disabilities rely on wheelchairs due to lower-limb paralysis. There is a substantial correlation between the level of upper-limb functional recovery and the quality of daily life. This study aimed to design and evaluate a device that utilizes various muscle contractions to enhance the effectiveness of upper-limb rehabilitation exercises. The results from the isometric performance assessment showed percentage error rates of >30% for 5–30 kg. Correction equations were employed, and the isometric performance assessment resulted in error rates below 2.1% for 5–30 kg. Isokinetic performance assessment using video analysis evaluated a consistent wire speed from 10 cm/s to 70 cm/s with an average error rate of 0.91% across all speeds. This study demonstrates the ability to accurately measure various muscle contractions and showcases the potential for real-time feedback. This highlights how such a device could be helpful for target groups, including older adults and individuals with disabilities, during upper-limb rehabilitation exercises.

## 1. Introduction

Advancements in cutting-edge science, technology, and healthcare have resulted in an aging population with many having extended average lifespans. Consequently, the healthcare market targeting older and disabled individuals is rapidly growing. Ongoing research is diligently exploring opportunities to improve health and the quality of life through the convergence of information communication and medical technologies. This includes the development of user-friendly rehabilitation medical devices, biometric signal measurement, analysis technologies, and personalized rehabilitation training software.

Muscle contraction enables bodily movements, and assessing muscle strength is crucial for evaluating muscle function and setting goals for strength improvement [1]. Muscle contractions can be categorized as isometric, isotonic, or isokinetic. Isokinetic contractions involve both the shortening and lengthening of the muscles [2]. These contractions are typically performed using expensive specialized equipment and expert personnel due to the unnatural nature of the movement for human performance. Consequently, individuals with physical disabilities face more significant challenges while performing isokinetic contractions [3]. Isometric muscle strength measurement using a load cell was applied to rugby players who were frequently concussed. To measure neck muscle strength, it was repeatedly measured using a load cell, and the reliability of the value was confirmed [4].

In contrast, stroke patients are known to exhibit a significant correlation between the level of upper-limb function recovery and the quality of daily life. Thus, the importance of upper-limb rehabilitation exercises has been emphasized [5]. The rehabilitation protocol includes acute, intermediate, and advanced strengthening and activity stages [6]. The acute phase focuses on reducing pain and inflammation; the intermediate stage aims to restore muscle balance; the advanced stage targets muscle strength and improving endurance; and the activity phase aims to enhance muscle power.

Isometric, isotonic, and isokinetic muscle contractions are utilized throughout the rehabilitation process, and the exercises progress from single- to multi-joint movements. An all-in-one device that can perform isometric, isotonic, and isokinetic muscle contractions while providing resistance variation through motor control will enhance the effectiveness of exercise rehabilitation in the general population. Additionally, the device will ensure stability and efficiency in rehabilitating older and disabled individuals. Furthermore, to objectively monitor precise movements and provide real-time feedback, applying motion-sensing technology in smart upper-limb rehabilitation devices would be beneficial [7].

This study aimed to design and evaluate a rehabilitation exercise device that enables isometric, isotonic, concentric, and isotonic eccentric movements with electronic load control, allowing for the programming of various load conditions and fluctuations. Our findings demonstrate the feasibility of accurately assessing various muscle contractions and the potential of motion-sensing technology to enhance rehabilitation exercises. The developed device shows promising implications for practical muscle strength training in diverse populations, including stroke patients and individuals with disabilities.

## 2. Materials and Methods

### 2.1. Rehabilitation Exercise Devices

#### 2.1.1. Design of Equipment and Sensor Configuration

**Figure 1:**
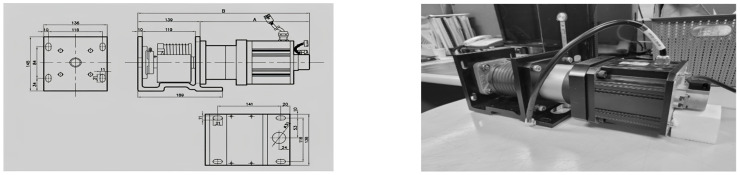
Design and fabrication of the electronic load control motor.

A rehabilitation exercise device was designed and fabricated to enable isometric, isotonic concentric, and isotonic eccentric movements. A motor was designed and constructed for electronic load control, allowing the programming of various load conditions and fluctuations (Figure 1). The specification for the motor is AC SERVO DRIVE HD-FD05AB-B4, and the manufacturer is ABLE (Shanghai, China). Specific specifications for this motor are described in Table 1.

**Figure 2:**
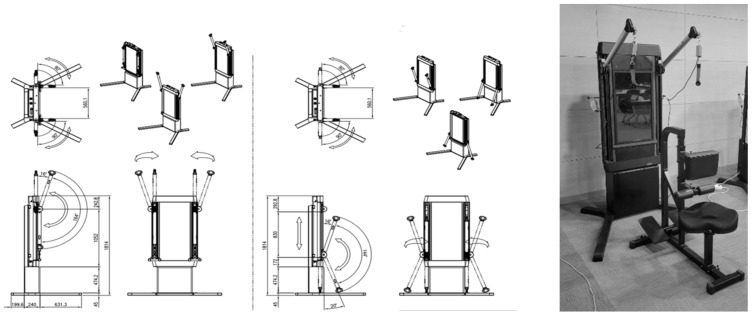
The upper-limb rehabilitation exercise device features an armrest with a rotation radius and specific product characteristics.

The rehabilitation exercise device was designed with an internal structure that allows the integration of mechanical components and a dedicated motor. The armrest attached to the mechanism has a total movement range of 1.05 m and can be adjusted in the vertical direction. The angle adjustments were 164° in the vertical direction and 90° in the horizontal direction. The device uses a wire connected to the armrest to perform upper-limb rehabilitation exercises. To measure the pulling speed and force applied to the wire by the user using both hands (left and right), the design incorporates Hall sensors and load cells (Figure 2).

#### 2.1.2. Module Composition

**Figure 3:**
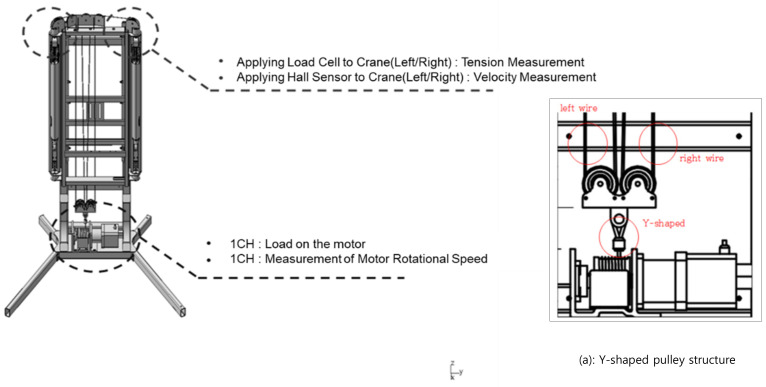
Arrangement of sensors to measure speed and force.

**Figure 4:**
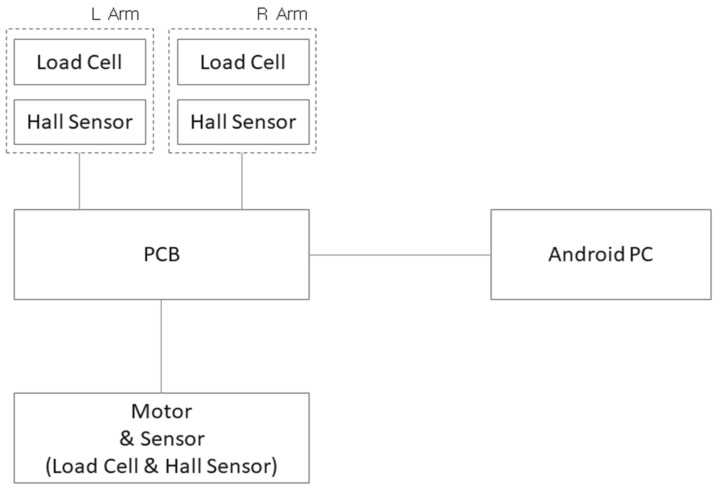
Electronic block diagram.

The measurement module of the rehabilitation exercise device consists of a load cell (SBA-200L, CAS, Seoul, Republic of Korea; measuring range 0–200 kg) and a Hall sensor (A3144, ALLEGRO, Manchester, MH, USA) to measure the force and speed, respectively, exerted by the user on both sides (left and right) (Figure 3). Using a Y-shaped pulley structure on a single motor ensures control adaptability even when different resistances are applied to the wires on the left and right sides (a in Figure 3). The load cell used for force measurement has a maximum weighing capacity of 200 kg. It is compact, with a thickness of only 20 mm, making it suitable for integration into the device. The collected and measured sensor values are processed and converted into weight and repetition counts using a microcontroller unit (ESP32, ESPRESSIF, Shanghai, China) integrated into the module. The collected data are then transmitted to the central unit of the exercise device using an RS232 communication module to provide exercise information (Figure 4).

For isometric exercise control, the applied force increases proportionally with the set load up to the set point. Beyond the set point, the force rapidly increases proportionately with the set load to restrict further displacement.

For isotonic exercise control, when force is applied to the stationary wire, the speed increases proportionally with the applied force until it reaches the set speed. To restrict further increases in the speed, the load increases accordingly. Isotonic exercise control is limited to concentric movements, while eccentric movements return to the set load.

For isotonic exercise control, the wire is controlled by the force applied in the rewinding direction based on the set load. This design allows pattern-specific force control during isotonic exercises. Isotonic exercise is divided into isotonic concentric movements, which are composed of pattern load variations based on a constant force and trajectory range, and isotonic eccentric movements, which consist of three components: movement, eccentric movement, and pattern loads.

### 2.2. Rehabilitation Exercise Information Analysis

#### 2.2.1. Acquisition of Weight Information from the Force Sensor

**Figure 5:**
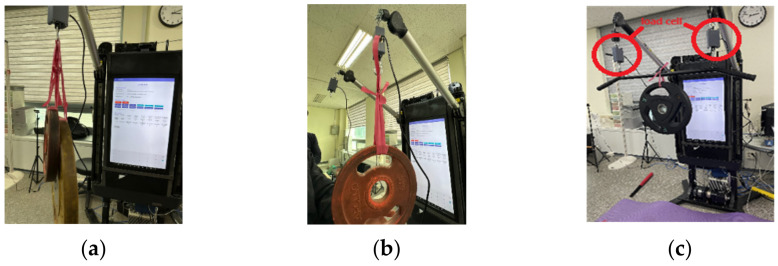
Weighting test. ((**a**): The wire on the left side. Left arm measurement. (**b**): The wire on the right side. Right arm measurement. (**c**): The wire on the left side(left red circle). Center measurement; The wire on the right side(right red circle). Center measurement.

To obtain weight information from the force sensor, the following method was employed. The internal motor outputs force values as electrical signals, which are then utilized by attaching a load cell to the external wire end of the device. This enables the measurement of the user’s force and the subsequent calculation of the corresponding weight (Figure 5). In order to address external isotonic resistance or provide specific isometric and isokinetic internal resistance, the Hall sensor within the motor detects the rotational state of the motor and converts it into electrical signals. Consequently, to verify the accurate recognition of electrical values corresponding to the correct weight, an external load cell was utilized for verifying the alignment of electrical values with the perceived weight. Next, the collected actual readings were converted into weight values by calibrating or applying conversion factors, thus obtaining weight measurements in units such as kilograms or pounds. Subsequently, the weight information from the hardware sensor was transmitted to a software application that processed and displayed it on a screen, acting as an interface for visualizing the weight information in a user-friendly manner.

#### 2.2.2. Weight Information Acquisition through a Software Algorithm

The values transmitted from the load cell printed circuit board (PCB) were processed and utilized to acquire weight information. The application programming interface for serial port communication was employed to initialize the load cell PCB variables and set up the input and output streams for communication with the PCB. After setting up the streams, the hardware data were detected. Once received, the data were read and stored in a buffer. The data stored in the buffer were then converted into a string format, which could be processed as “$1, 2, 3, 4, 5, 6”. By interpreting this string, the unique character after “$” represents the type of data: the value at position 1 represents the left rotation count; the value at position 2 represents the correct rotation count; the value at position 3 represents the speed; the value at position 4 represents the total load; the value at position 5 represents the left-side load; and the value at position 6 represents the right-side load.

The weight information can be obtained by processing and separating the formatted string. Furthermore, the final weight can be determined by multiplying the obtained weight information by the constant values obtained through the experiments.

### 2.3. Experiments

To validate the accuracy of the weight measurements in the upper-limb rehabilitation exercise device, performance evaluation experiments were conducted by differentiating between fixed (isometric) and unfixed wire conditions (isotonic and isokinetic).

The performance-evaluation experiment involved attaching weights of 5, 10, 20, and 30 kg to the left, right, and midpoint armrests. The weights were measured repeatedly, and the values obtained from the developed device were analyzed (Figure 5). Throughout the experiment, the armrest positions and angles were maintained consistently, with a height level of 3 (range 1–9), vertical angle of 20°, and horizontal angle of 90°for all weight measurements. This standardization ensured that the weight measurements were conducted under controlled and comparable conditions.

### 2.4. Statistical Analysis

The test–retest reliability of the data collected was analyzed, and the Pearson correlation coefficient (r) was used for calculating the concurrent validity using the SPSS Package v.20 (IBM Corp., Armonk, NY, USA). The test–retest reliability of the consistency of weight and velocity measurements was performed using an average error ratio. To investigate the validation between the two devices, we used the Bland–Altman method (*p* < 0.05). The limits of agreement were set to ±1.96 standard deviations from the mean.

## 3. Results

### 3.1. Isometric Performance Evaluation

During the isometric performance evaluation experiment, the average error rates were 33.7% for a 5 kg load, 37.0% for a 10 kg load, 33.6% for a 20 kg load, and 34.6% for a 30 kg load (Table 2). To correct for the isometric error, the following formula was employed:

Compensation formula: motor value − (0.313 × motor value) + 0.042

### 3.2. Isotonic Performance Evaluation

The isotonic performance was evaluated by applying the calibration formula derived from the isometric experiments in which the wire was fixed. The isotonic performance was assessed by verifying whether the predetermined weights were consistently measured in each wire segment without controlling the speed. The wire was divided into four segments based on the length ratio, and the values measured for each segment were analyzed. The predetermined weight loads were set to 5, 10, 20, and 30 kg, identical to those used in the isometric experiments. The average error rate was below 2.1% for all loads, confirming that the adjusted formula was acceptable (Table 3).

### 3.3. Isokinetic Performance Evaluation

Isokinetic performance evaluation was conducted with a 30 kg weight attached, maintaining a constant wire speed of 10, 20, 30, 40, 50, 60, and 70 cm/s. The video analysis was conducted at a frame rate of 60 Hz with a time interval of 0.01667 s. The wire was divided into four segments (each 20 cm) to assess the maintenance of a consistent speed in each segment. The analysis was conducted at a time interval of 0.01 s.

**Figure 6:**
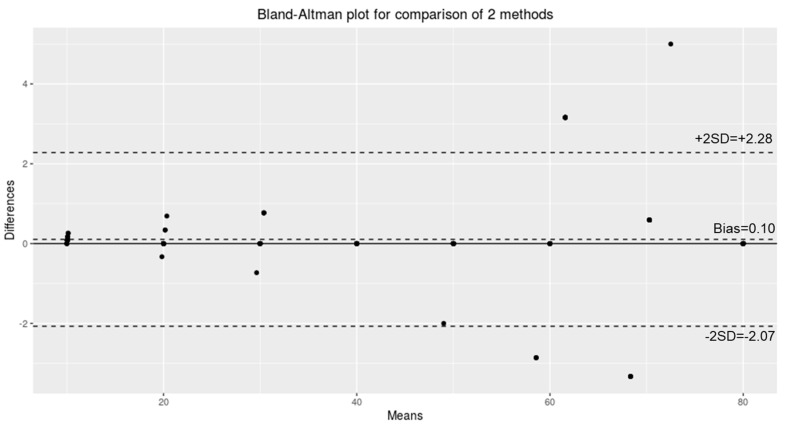
Bland-Altman agreement and limits of agreement.

For a detailed analysis, the frame rate per second was extracted for each segment from the recorded video, and the actual time was calculated. The actual time was then divided by the segment distance (20 cm) to calculate the speed. The results showed an average error rate of 0.91% for all the loads, indicating that the isokinetic performance was satisfactory (Figure 6).

## 4. Discussion

This study aimed to evaluate the level of muscle strength measured through isometric, isotonic, and isokinetic muscle contractions using a load cell in an upper-limb rehabilitation device. Additionally, it sought to assess the reliability of the recorded data. A commercially available standard laboratory setting for muscle strength measurement was used as a reference for the experiment. The methods employed in this study were as follows:

(1) Isometric strength evaluation: To verify the reliability of the isometric strength assessment, weights were attached to the cable connected to the motor. The measured values obtained through the load cell were repeatedly recorded to confirm the reliability of the isometric measurement.
(2) Isotonic strength evaluation: To verify the reliability of the isotonic strength assessment, weights were attached to the cable connected to the motor, and the load cell data were measured during the descent phase at intervals. The data were evaluated repeatedly to confirm the reliability of the isotonic measurement.
(3) Isokinetic strength evaluation: To verify the reliability of the isokinetic strength assessment, weights were attached to the cable connected to the motor, and a video was recorded at 60 frames per second during descent, ensuring that the motor maintained a constant speed during the fall.

Isometric, isotonic, and isokinetic muscle contractions are used in the rehabilitation of musculoskeletal disorders. For upper-limb rehabilitation, electromyographic analysis of the anterior deltoid and pectoralis major muscles during horizontal abduction with a resistance band attached to the arm showed increased activation of the anterior deltoid but decreased activation of the pectoralis major muscle [8]. In the rehabilitation process for patients with shoulder impingement syndrome using dumbbells, the evaluation of muscle activation showed increased activation of the lower trapezius and anterior deltoid muscles [9]. Additionally, applying 5 s maximum isometric contractions (1RM: Repetition Maximum 80–85%) twice a week for 8 months to individuals with osteoporosis increased bone density in the femoral neck and lumbar spine. Furthermore, training with a load cell myometer at intensities of 60–90% of the maximum isometric contraction during arm curl exercise increased the muscle cross-sectional area and maximum isometric contraction strength [10].

Isometric strength measurements are applicable in individuals with physical disabilities. Meta-analyses have used data from studies employing portable isometric dynamometry to assess upper-limb strength in children with cerebral palsy [11]. It is said that it is useful and reliable even referring to a study that measured the isometric strength of the elderly using a hand-held dynamometer [12]. The portable hand-held dynamometer has the advantage of being inexpensive, but it cannot measure motion isotonic or isokinetic muscle function. Since it is more important for human muscles to exhibit muscle functions in movement in daily life, it is important to evaluate muscle strength in isotonic or isokinetic muscle contraction. From this point of view, it has the advantage of being able to measure and train isometric, isotonic, and isokinetic muscle contractions while being cheaper than general isokinetic equipment. However, this device has the disadvantage that a device that fixes the axis for single-joint movement has not yet been developed separately.

As a result of measuring the strength of the equipment using the load cell, the portable dynamometer, and the upper and lower limbs, there was a significant correlation. Through these results, it can be seen that the equipment of this study using load cells can also be applied to measure muscle strength [13]. The use of isometric contractions facilitates the evaluation of muscle strength and promotes safe and effective strength training.

Various tools, such as resistance bands, free weights, and weight machines, are commonly used for training involving isotonic contractions. Applying isotonic training three times a week for 6 weeks to stroke patients has been shown to result in improved maximum strength, and this effect persists for up to 9 months [14]. Additionally, patients with Parkinson’s disease show reduced tremors after medium-intensity upper-limb isotonic strength training [15].

Isokinetic contractions are often used to rehabilitate single-joint movements. Patients with shoulder joint and anterior instability symptoms were subjected to isokinetic rehabilitation programs with varying angular velocities (from 30°/s to 300°/s in 30°/s increments) [16]. The provision of shoulder external and internal isokinetic training to tennis players three times a week for 6 weeks, at angular velocities of 90°/s, 120°/s, 150°/s, and 180°/s, resulted in increased muscle strength, serving speed, and endurance [17].

Isokinetic training can also be applied to individuals with disabilities. In one study, it was conducted using the CON-TREX isokinetic equipment (Physiomed Elektromedizin AG, Schnaittach, Germany) for 45 min, three times a week, over a 6-week period. Although no significant improvements were observed, the functional abilities of stroke patients did not decline and remained significant [18].

## 5. Conclusions

This upper-limb rehabilitation device that was developed can be used for maximum isometric strength measurement and enables real-time monitoring of changes in isometric strength, thereby facilitating practical muscle strength training. This study evaluated the reliability of measurements for maximum isometric, isotonic, and isokinetic contractions in upper-limb rehabilitation devices using a load cell. The results demonstrated the feasibility of measuring various muscle contractions and reliably assessing muscle strength. Additionally, an intelligent upper-limb rehabilitation device with motion-sensing technology was shown to provide real-time feedback and accurate movement monitoring, thereby enhancing the effectiveness of rehabilitation exercises.

The findings of this study offer important prospects in an aging society with an increasing demand for healthcare services targeting older and disabled individuals. By integrating information communication and medical technologies, innovative rehabilitation devices tailored to individual needs can contribute to the optimization of patient outcomes.

Based on these research findings, several recommendations are proposed:

(1) Further research: Additional research is required to explore the long-term effects of rehabilitation programs utilizing isometric, isotonic, and isokinetic muscle contractions in specific patient populations, including those with musculoskeletal disorders or neurological conditions.
(2) Development of customized rehabilitation plans: Healthcare professionals should establish personalized rehabilitation plans that consider each patient’s unique needs and conditions. Smart rehabilitation devices that offer real-time feedback can facilitate personalized exercise regimens and effectively monitor progress.
(3) Collaboration and education: Collaboration among researchers, technology developers, healthcare professionals, and policymakers can foster an innovative environment and the integration of rehabilitation technologies. Adequate education and training regarding the use of advanced rehabilitation devices are necessary for healthcare professionals.

In summary, developing upper-limb rehabilitation devices that integrate information and medical technology opens up new possibilities for improving patient care and optimizing rehabilitation program outcomes in musculoskeletal rehabilitation. The utilization of these devices has the potential to enhance the quality of life of patients with musculoskeletal disorders and neurological conditions.

## 6. Patents

(1) Exercise equipment that supports three exercise methods, Patent Application Number: 10-2022-0087698 (http://www.kipris.or.kr/)
(2) Rehabilitation exercise aid equipment, Patent Application Number: 10-2023-0038409 (http://www.kipris.or.kr/)
(3) Rehabilitation exercise aid equipment, Patent Application Number: 10-2023-0038420 (http://www.kipris.or.kr/)

## Figures and Tables

**Table 1:** Specifications of the motor.

Rated Torque	N·m	6.2
	kg·cm	63.3
Peak Torque	N·m	18.6
	kg·cm	190
Rated Speed	r/min	1000
Peak Speed	r/min	2000
Gear Ratio	-	1/10
Cable Force	kg	150
Friction Force	kg	10

**Table 2:** Wire fixed (isometric) measurement result and error rate.

		Actual Weight: 5 kg				Actual Weight: 10 kg				Actual Weight: 20 kg				Actual Weight: 30 kg			
		Left ^a^	Right ^b^	Middle Left ^c^	Middle Right ^d^	Left	Right	Middle Left	Middle Right	Left	Right	Middle Left	Middle Right	Left	Right	Middle Left	Middle Right
Measured weight	Trial 1	7.29	7.23	3.66	3.54	14.99	14.88	7.59	7.35	29.07	28.85	14.67	14.29	44.08	43.44	22.19	21.70
	Trial 2	7.29	7.23	3.70	3.50	14.99	14.88	7.65	7.29	29.05	28.86	14.65	14.31	44.03	43.51	22.32	21.58
	Trial 3	7.29	7.23	3.67	3.58	14.98	14.89	7.60	7.33	29.07	28.86	14.73	14.23	44.04	43.71	22.49	21.41
Measured average weight (kg)		7.29	7.23	3.68	3.54	14.99	14.88	7.61	7.32	29.06	28.86	14.68	14.28	44.05	43.55	22.33	21.56
Measured error rate (%)		45.8	44.6	23.6	20.8	49.9	48.8	26.1	23.2	45.3	44.3	23.4	21.4	46.8	45.2	24.4	21.9
Measured average error ratio (%)		33.7				37.0				33.6				34.6			
After applying compensation formula (kg)		5.05	5.01	2.57	2.47	10.34	10.26	5.27	5.07	20.01	19.87	10.13	9.85	30.30	29.96	15.38	14.85

^a^: The wire on the left side in Figure 5. Left arm measurement. ^b^: The wire on the right side in Figure 5. Right arm measurement. ^c^: The wire on the left side in Figure 5. Center measurement. ^d^: The wire on the right side in Figure 5. Center measurement.

**Table 3:** Wireless unsecured (isotonic) measurement results and error rate.

Wire Position	5 kg								10 kg								20 kg								30 kg							
	Section 1		Section 2		Section 3		Section 4		Section 1		Section 2		Section 3		Section 4		Section 1		Section 2		Section 3		Section 4		Section 1		Section 2		Section 3		Section 4	
	L	R	L	R	L	R	L	R	L	R	L	R	L	R	L	R	L	R	L	R	L	R	L	R	L	R	L	R	L	R	L	R
1st	4.91	4.91	4.95	4.95	4.99	4.99	4.99	4.99	9.90	10.19	9.89	10.25	9.93	10.31	9.97	10.29	20.41	20.38	20.25	20.38	20.35	20.44	19.58	20.38	30.63	30.52	31.10	30.68	-	30.48	-	30.58
2nd	4.95	4.95	4.98	4.98	5.00	5.00	5.01	5.01	9.84	10.24	9.90	10.28	9.62	10.32	9.95	10.34	20.71	20.56	20.16	20.71	20.30	20.75	20.04	20.73	30.63	30.37	30.94	30.60	30.91	30.61	30.50	30.06
3rd	4.95	4.95	4.68	4.68	4.99	4.99	5.01	5.01	9.82	10.50	9.90	10.22	9.95	10.32	9.93	10.17	20.03	20.51	20.89	20.68	20.32	20.95	20.12	20.70	30.54	30.34	30.74	31.51	30.85	30.61	30.79	30.10
4th	4.95	4.95	4.99	4.99	5.00	5.00	5.01	5.01	9.86	10.21	10.25	10.26	9.97	10.29	9.22	10.28	19.98	20.51	20.13	21.11	20.24	20.73	19.67	20.09	30.52	30.52	30.69	30.74	30.80	30.70	30.62	30.53
5th	4.95	4.95	4.99	4.99	5.01	5.01	4.44	4.44	9.83	10.03	9.91	10.35	9.95	10.07	9.79	9.45	20.00	20.35	20.14	20.54	20.25	20.56	19.52	20.12	30.61	30.45	30.78	30.64	31.27	30.77	30.86	30.32
6th	4.95	4.95	4.99	4.99	5.00	5.00	4.43	4.43	9.82	10.24	9.91	10.24	9.54	10.29	9.42	10.31	20.00	20.53	20.18	21.19	20.29	20.73	19.98	20.14	30.54	30.56	30.70	30.71	30.76	30.78	30.43	30.26
7th	4.95	4.95	4.99	4.99	4.99	4.99	4.44	4.44	9.83	10.07	9.91	10.08	9.96	10.14	9.81	9.89	20.06	20.55	20.21	20.66	20.29	20.71	19.49	20.17	30.69	30.41	30.85	30.54	30.85	30.69	30.66	30.63
8th	4.95	4.95	4.98	4.98	4.99	4.99	4.44	4.44	9.83	10.21	9.91	10.26	9.96	10.30	9.77	10.08	19.98	20.57	20.84	20.68	20.28	20.70	19.93	20.65	30.61	29.96	30.82	31.15	31.20	30.25	30.70	30.30
9th	4.95	4.95	4.97	4.97	4.99	4.99	4.44	4.44	9.82	10.22	9.89	10.19	9.94	10.30	9.95	9.60	20.75	20.52	20.11	20.68	20.29	20.75	19.74	20.64	30.53	29.96	30.79	30.12	30.96	30.22	30.43	29.60
10th	5.25	5.25	4.95	4.95	4.99	4.99	4.47	4.47	9.82	10.18	9.90	10.25	9.97	10.29	9.95	9.68	20.05	20.51	20.21	20.70	20.28	20.73	19.48	20.29	30.80	29.77	30.91	30.06	30.91	30.26	30.76	30.08
Average error (kg)	0.02	0.02	0.05	0.05	0.00	0.00	0.33	0.33	0.16	0.21	0.06	0.24	0.12	0.26	0.22	0.01	0.20	0.50	0.31	0.73	0.29	0.71	0.25	0.39	0.61	0.29	0.83	0.68	0.95	0.54	0.64	0.25
Error rate (%)	0.5	0.5	1.1	1.1	0.1	0.1	6.6	6.6	1.6	2.1	0.6	2.4	1.2	2.6	2.2	0.1	1.0	2.5	1.6	3.7	1.4	3.5	1.2	2.0	2.0	1.0	2.8	2.3	3.2	1.8	2.1	0.8
Average error ratio (%)	2.1								1.6								2.1								2.0							

## Data Availability

Data are contained within the article.
